# Altered Cohesin Gene Dosage Affects Mammalian Meiotic Chromosome Structure and Behavior

**DOI:** 10.1371/journal.pgen.1003241

**Published:** 2013-02-07

**Authors:** Brenda Murdoch, Nichole Owen, Michelle Stevense, Helen Smith, So Nagaoka, Terry Hassold, Michael McKay, Huiling Xu, Jun Fu, Ekaterina Revenkova, Rolf Jessberger, Patricia Hunt

**Affiliations:** 1 School of Molecular Biosciences, Washington State University, Pullman, Washington, United States of America; 2 Institute of Physiological Chemistry, Technische Universität Dresden, Dresden, Germany; 3 Sydney University and the North Coast Cancer Institute, Lismore, New South Wales, Australia; 4 Divisions of Research and Radiation Oncology, Peter MacCallum Cancer Centre, East Melbourne, Victoria, Australia; 5 Genomics, BioTec, Technische Universität Dresden, Dresden, Germany; 6 Department of Developmental and Regenerative Biology, Mount Sinai School of Medicine, New York, New York, United States of America; Stowers Institute for Medical Research, United States of America

## Abstract

Based on studies in mice and humans, cohesin loss from chromosomes during the period of protracted meiotic arrest appears to play a major role in chromosome segregation errors during female meiosis. In mice, mutations in meiosis-specific cohesin genes cause meiotic disturbances and infertility. However, the more clinically relevant situation, heterozygosity for mutations in these genes, has not been evaluated. We report here evidence from the mouse that partial loss of gene function for either *Smc1b* or *Rec8* causes perturbations in the formation of the synaptonemal complex (SC) and affects both synapsis and recombination between homologs during meiotic prophase. Importantly, these defects increase the frequency of chromosomally abnormal eggs in the adult female. These findings have important implications for humans: they suggest that women who carry mutations or variants that affect cohesin function have an elevated risk of aneuploid pregnancies and may even be at increased risk of transmitting structural chromosome abnormalities.

## Author Summary

Chromosome segregation errors during meiosis are the leading cause of birth defects and miscarriages in humans. While the basis for these errors is unknown, recent studies suggest that defective sister chromatid cohesion may be an important contributor. Accordingly, we tested the hypothesis that partial loss of gene function for either of two meiosis-specific cohesins, *Smc1b* or *Rec8*, might adversely affect synapsis or recombination between homologs during meiotic prophase. Our analyses of different mouse models demonstrate cohesin dosage effects on meiosis in both males and females. Importantly, reduced gene function led to an increase the frequency of chromosomally abnormal eggs in the adult female, suggesting that, in humans, women carrying cohesin mutations may be at an increased risk of chromosomally abnormal pregnancies.

## Introduction

In humans, the likelihood of an aneuploid conception is extremely high due to errors in chromosome segregation that occur during the meiotic divisions (reviewed in: [1]–[4]). Although errors occur during both spermatogenesis and oogenesis, over 90% of aneuploidy arises in the oocyte, and the incidence of errors is strongly enhanced by maternal age [1]. Recently, studies from several different laboratories have provided direct evidence that perturbations in cohesin proteins affect the orderly segregation of homologs at meiosis I (MI) and of sister chromatids at the second meiotic division (MII) [5]–[8] and deterioration of cohesion has been postulated to be a major mechanism of human age-related aneuploidy [7], [9], [10].

The trimeric core complex of cohesin is a heterodimer of SMC3 and SMC1 proteins that forms a two-sided triangle closed by a kleisin protein (reviewed in: [11]–[14]). Although vertebrates have a single SMC3 protein, there are two SMC1 variants (α and β) and one (SMC1β) is specific to meiocytes. Meiotic cells also have three kleisins (RAD21, RAD21L and REC8) that differ in their spatiotemporal features. The final cohesin component is a stromal antigen (SA) protein; two SA proteins are found in somatic cells (SA1 and SA2), a third, SA3 (STAG3), is present in meiocytes. Different combinations of core component proteins create a variety of cohesin complexes in vertebrate meiocytes [15], [16], but the particular functions of individual complexes remain poorly understood.

Mice deficient for either REC8 or SMC1β reveal essential meiotic roles for these cohesins. In both *Rec8^−/−^* and *Smc1b^−/−^* males, synaptonemal complexes (SC) are shortened, synapsis between homologous chromosomes is impaired, and spermatocytes die in early/mid pachytene [17]–[19]. In females, similar synaptic defects are evident in REC8-deficient oocytes, and cell death occurs around the time of dicytate arrest [17], . In females deficient for SMC1β, however, mature oocytes are produced (albeit at reduced numbers), levels of recombination are reduced and, importantly, sister chromatid cohesion (SCC) is poorly maintained and connections between homologs and sister centromeres are lost prematurely [18]. The report that these cohesion defects were remarkably elevated in 2- and 4- by comparison with 1-month old females provided the first evidence of an age-related weakening of cohesion in female mice [5].

More recently, a link between cohesins and age-related aneuploidy in normal female mice has been provided [7], [8], leading to the provocative hypothesis that deterioration of cohesins is the cause of the maternal age effect on aneuploidy [7]. However, although the human data suggest that loss of cohesin is a major factor, the available evidence suggests that multiple factors contribute to the age-related increase in segregation errors during human female meiosis (reviewed in: [4]).

The loss of cohesin hypothesis presupposes no or insufficient turnover of proteins in the cohesin complex after they are loaded onto chromosomes during prophase in the fetal ovary. Consistent with this idea, although meiosis-specific cohesins are transcribed during oocyte growth in the adult ovary [20], [21], there is no evidence that functional proteins are produced. Further, two lines of evidence suggest that the protein complex established during fetal development is both necessary and sufficient. First, if transcription of *Smc1b* is prevented in growing oocytes, chromosome segregation occurs normally [20], indicating that cohesin loaded during fetal development is sufficient for proper chromosome disjunction. Second, loss of cohesion induced by destruction of REC8 protein could not be rescued by ectopic expression of a *Rec8* transgene during oocyte growth [22].

The combined data from these recent studies in mice not only suggest that loss of cohesin plays a major role in meiotic errors, they imply that certain levels of cohesin must be maintained for proper chromosome segregation in oocytes. This, coupled with data from studies in Drosophila where reduction of the *Smc1* cohesin protein was used to increase nondisjunction in experimentally aged oocytes [23], caused us to wonder whether haploinsufficiency for meiosis-specific cohesin genes might induce an age-*independent* meiotic phenotype in mice. We report here evidence from studies using several different mouse models that partial loss of gene function for either *Smc1b* or *Rec8* results in perturbations in the formation of the synaptonemal complex (SC) that affect both synapsis and recombination between homologs during meiotic prophase. Importantly, these subtle prophase defects increase the frequency of eggs with chromosome abnormalities in the adult female. These findings have important clinical implications since they suggest that women carrying mutations or variants in meiosis-specific cohesin genes that affect cohesin dosage may be at increased risk of producing children with chromosome abnormalities.

## Results

### Cohesin heterozygotes have increased synaptic defects and decreased recombination levels

**Figure 1:**
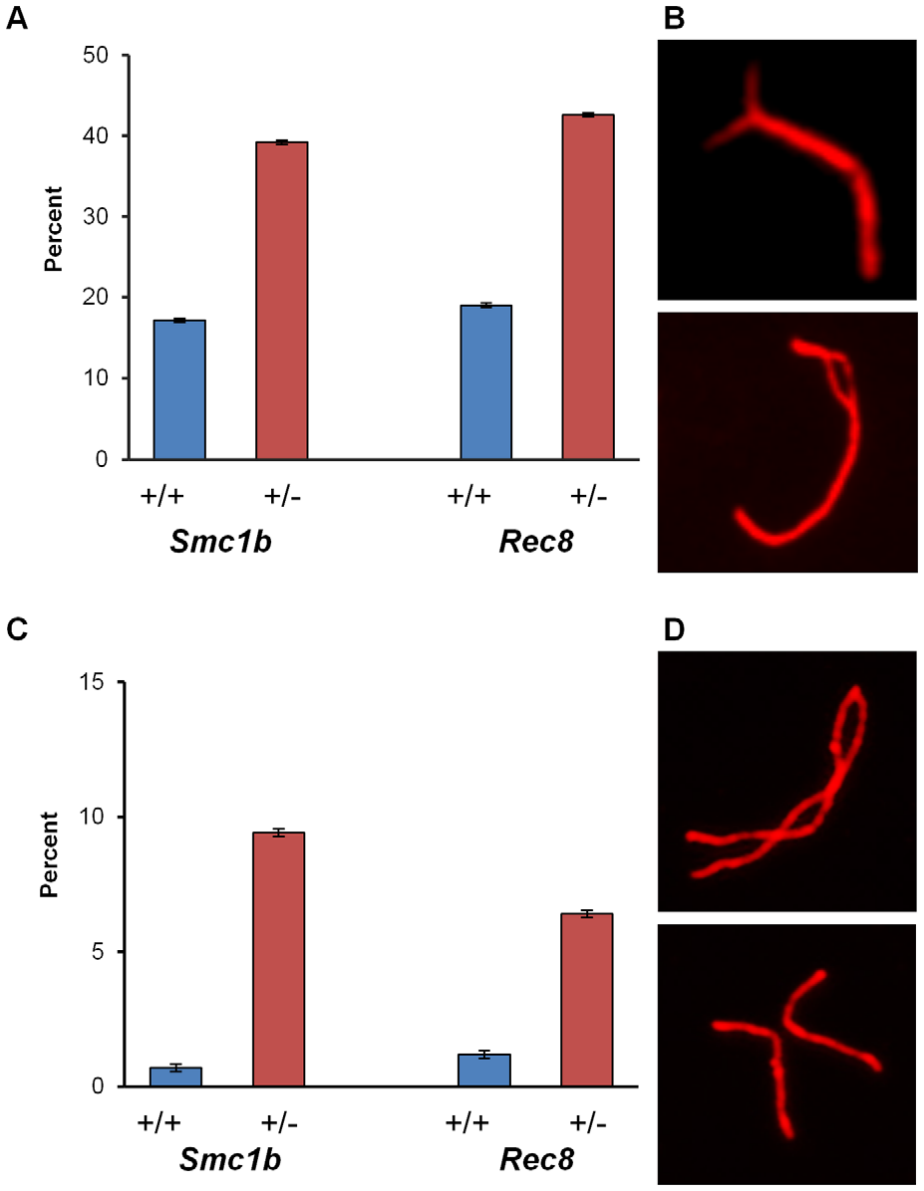
Synaptic errors are increased in cohesin heterozygotes. (A–D) Proportion of pachytene stage cells exhibiting minor and major synaptic defects. SCs were visualized using an antibody against SYCP3 to detect the axial/lateral elements of the synaptonemal complex. For *Smc1b*, n = 350 for heterozygous and n = 300 for wild-type siblings; for *Rec8*, n = 450 for heterozygous and n = 250 for wild-type siblings. (A) Proportion of cells (± SE) exhibiting minor defects. (B) Representative images of SCs with minor defects; SC with a fork (top), SC with an internal bubble (bottom). (C) Proportion of cells (± SE) exhibiting major synaptic defects. (D) Representative images of SCs with major defects; partial asynapsis (top), complete asynapsis (bottom).

In initial studies, we examined pachytene cells from females heterozygous for mutations in either *Smc1b* or *Rec8*. We analyzed the incidence of synaptic defects and recombination levels for each of the two cohesins, and observed significantly increased levels of defects in the heterozygotes. For the analysis of synapsis, we defined two broad categories of defects – minor or major – depending on the type and extent of the abnormality (see Materials and Methods and Figure 1). Minor defects were significantly increased in both *Smc1b* (35.7% of oocytes by comparison with 17.0% in sibling controls; χ^2^_1 df_ = 37.7; *p*<0.0001) and *Rec8* heterozygotes (43.1% of oocytes by comparison with 15.2% in controls; χ^2^_1 df_ = 64.7; *p*<0.0001) (Figure 1A). The most common minor defects observed in both *Smc1b* and *Rec8* heterozygotes were forks at the ends of the SC (Figure 1B, top panel). Major defects included pachytene oocytes with partial or complete asynapsis of at least one bivalent (Figure 1C, 1D). For *Smc1b* these defects were observed in 9.4% of oocytes from heterozygotes and only 0.3% from controls (χ^2^_1 df_ = 36.1; *p*<0.0001); for *Rec8*, 6.4% of oocytes from heterozygotes had major defects compared to only 1.2% from controls (χ^2^_1 df_ = 17.9; *p*<0.0001).

**Figure 2:**
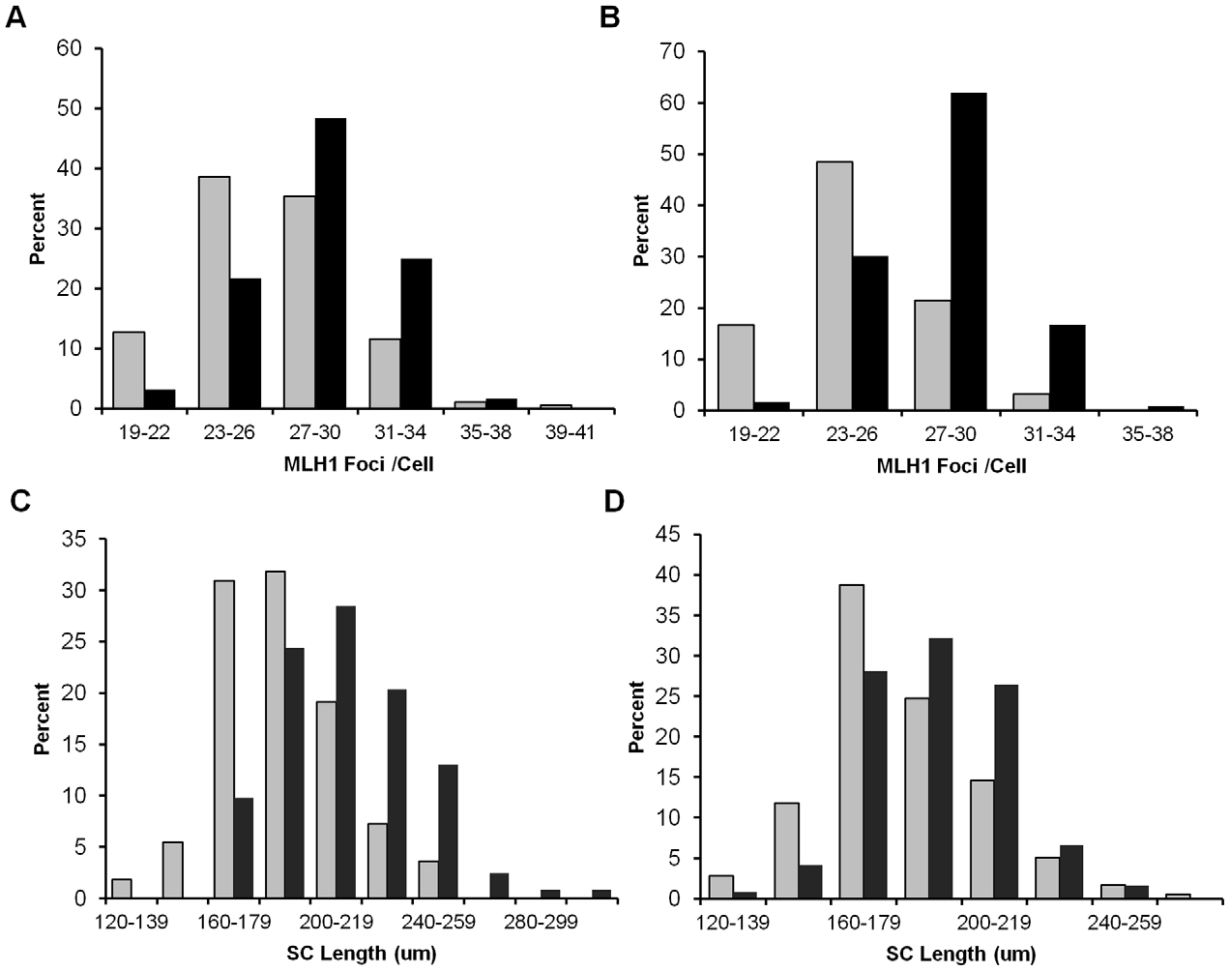
Recombination levels are reduced in cohesion heterozygotes. (A) The number of MLH1 foci in pachytene cells from *Smc1b* heterozygotes was significantly decreased by comparison with wild-type controls (mean MLH1 foci/cell ± SE = 25.0±0.25 and 27.9±0.25, respectively; t = 8.2; *p*<0.0001). Data represent 126 cells from 5 heterozygous females (grey bars) and 113 cells from 5 wild-type siblings (black bars). (B) A similar reduction was evident in *Rec8* heterozygotes (mean MLH1 foci/cell ± SE = 26.6±0.26 and 28.7±0.28, respectively; t = 5.3; *p*<0.0001). Data represent 181 cells from 7 heterozygotes (grey bars) and 124 cells from 5 sibling controls (black bars). (C, D) SC length was also significantly decreased for both (C) *Smc1b* (mean ± SE = 186.7±1.18 µm for 126 cells from 5 heterozygotes (grey bars) and 211.7±1.05 µm for 113 cells from 5 controls (black bars); t = 7.2; *p*<0.0001) and (D) *Rec8* heterozygotes (181.1±0.96 µm for 179 cells from 7 heterozygotes (grey bars) and 194.3±1.14 µm for 123 cells from 5 controls (black bars); t = 3.1; *p*<0.001).

To determine if recombination levels were affected, we analyzed MLH1 foci, since this mismatch repair protein localizes to sites of future crossovers [24]. The mean number of foci per cell was significantly reduced in both *Smc1b* and *Rec8* heterozygotes (Figure 2): for *Smc1b*, means ± standard errors were 27.9±0.25 and 25.0±0.25 for controls and heterozygotes, respectively (t = 8.2; *p*<0.0001) and, for *Rec8*, 28.7±0.28 and 26.6±0.26 for controls and heterozygotes, respectively (t = 5.3; *p*<0.0001). Because recombination failure is a well-known correlate of meiotic nondisjunction [2], [4], we examined the frequency of SCs lacking an MLH1 focus. As shown in Table 1, a striking difference in the proportion of SCs with 0, 1, 2 or 3 or more MLH1 foci was evident in both *Smc1b* and *Rec8* heterozygotes, with the frequency of “MLH1-less” SCs highly elevated in both (χ^2^_1 df_ = 12.6, *p*<0.001 and χ^2^_1 df_ = 16.6, *p*<0.001, respectively). In addition to comparing the total number of SCs involved, we compared the incidence of cells with one or more SC lacking a focus and found a significant increase in such cells in both heterozygotes: 55 of the 113 cells (49%) examined from *Smc1b* heterozygous females by comparison with 36 of the 126 cells (29%) in wild-type controls (χ^2^_1 df_ = 4.1; *p*<0.05); and 58 of the 181 cells (32%) examined from *Rec8* heterozygotes by comparison with 12 of the 123 cells (10%) in controls (χ^2^_1 df_ = 12.4; *p*<0.001).

**Table 1:** Comparison of MLH1 foci distribution in oocytes from cohesin heterozygotes and sibling controls.

Genotype	Foci per SC				TotalSCs
	0	1	2	≥3	
*Smc1b* +/+	36 (1.4%)	1512 (60.0%)	913 (36.2%)	59 (2.3%)	2520
+/−	67 (3.0%)	1590 (70.4%)	574 (25.4%)	29 (1.3%)	2260
*Rec8* +/+	13 (1.0%)	1436 (58.4%)	971 (39.5%)	50 (2.0%)	2460
+/−	70 (2.0%)	2323 (64.2%)	1186 (32.8%)	41 (1.1%)	3620

Although the focus of our studies was on oogenesis, we conducted an analysis of pachytene cells in males to determine if the phenotypic consequences of cohesin heterozygosity extend to spermatogenesis. Similar effects on recombination were evident in male carriers of both cohesin mutations (Table 2); e.g., mean MLH1 values per cell were significantly decreased for both *Smc1b* (t = 7.3; *p*<0.0001) and *Rec8* (t = 6.8; *p*<0.0001) heterozygotes by comparison with wild-type males.

**Table 2:** Mean MLH1 foci/cell in male heterozygotes and wild-type siblings.*

Genotype	Number of cells	Average MLH1
*Smc1b* +/+ *Rec8* +/+	87	22.3±0.24
*Smc1b* +/−	95	20.3±0.17
*Rec8* +/−	56	20.4±0.20

* Matings of compound heterozygotes were used to generate males for these studies.

### Synaptonemal complex morphology is altered in cohesin heterozygotes

**Figure 3:**
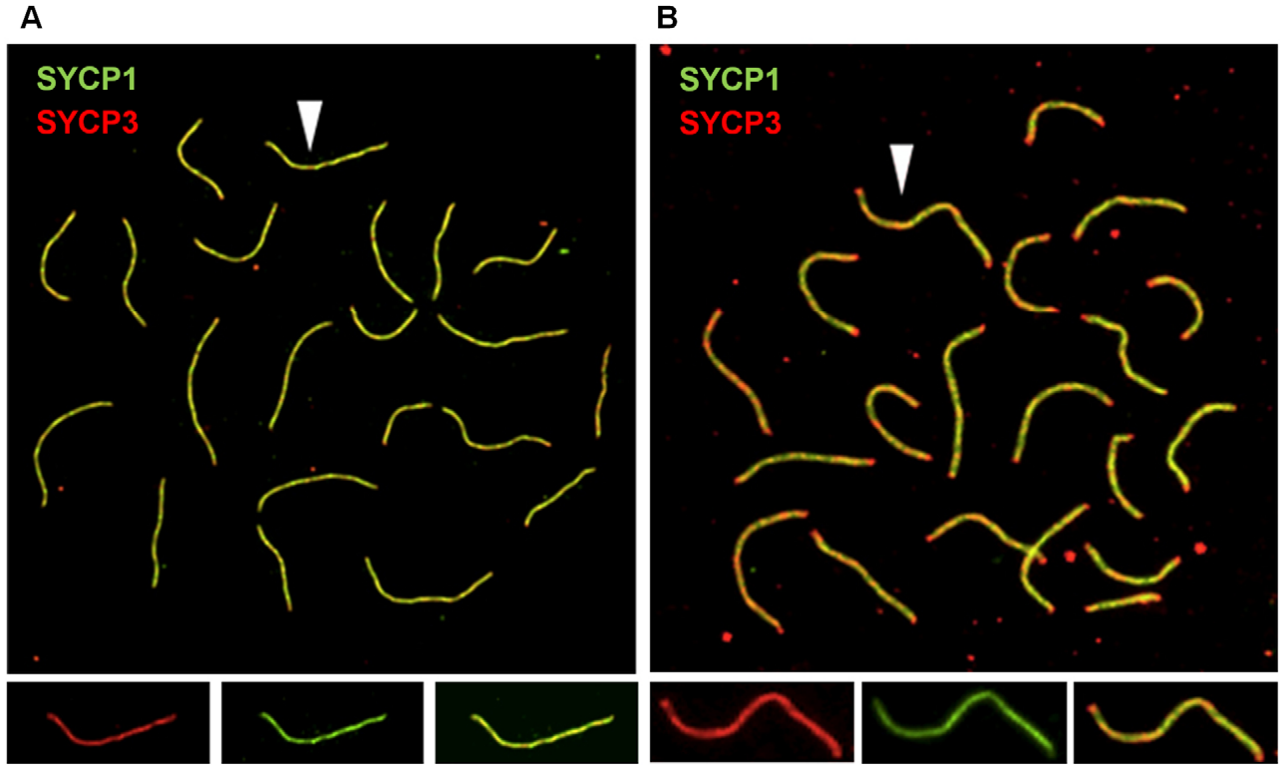
Synaptonemal complex formation is disturbed in cohesin heterozygotes. (A, B) Representative images of pachytene stage oocytes from control and *Rec8* heterozygous females. SCs were visualized using antibodies against SYCP1, to detect the transverse filament of the central element (green), and SYCP3, to detect the axial/lateral elements (red). (A) Pachytene cell from wild-type female; arrow denotes single SC shown in enlarged images below showing SYCP3 signal (left panel), SYCP1 signal (middle panel) and merged signals (right panel). (B) Pachytene cell from a *Rec8* heterozygote showing non-uniform SC staining, with red staining at the ends of most SCs; arrow denotes single SC shown in enlarged images below showing SYCP3 (left panel), SYCP1 (middle panel) and merged (right panel) signals.

Because the number of MLH1 foci is correlated with SC length [25], we measured the SCs in pachytene stage cells (Figure 2C, 2D). The mean total SC length was significantly reduced in oocytes from both *Smc1b* (186.7±1.18 µm and 211.7±1.05 µm, for heterozygotes and controls, respectively; t = 7.2; *p*<0.0001) and *Rec8* heterozygotes (181.1±0.96 µm and 194.3±1.14 µm for heterozygotes and controls, respectively; t = 3.1; *p*<0.001). Importantly, the overall reduction in genome-wide SC length (10% in *Smc1b* and 6% in *Rec8* heterozygotes) was proportional to the decrease in mean MLH1 counts. Further, we noted a difference in SC morphology in oocytes from heterozygotes by comparison with wild-type siblings. Specifically, dual staining with antibodies to SYCP3, which detects the axial/lateral elements, and SYCP1, which detects the transverse filament of the SC, typically yields a merged yellow signal, indicating co-localization of the two proteins. However, in both *Smc1b* and *Rec8* heterozygotes, SCs exhibited a harlequin appearance, with regions of discontinuous red and green signals as well as merged yellow signals. This was particularly evident in telomeric regions (Figure 3A, 3B).

### Synaptic and recombination defects are also evident in homozygous carriers of an *Smc1b* hypomorphic mutation

**Figure 4:**
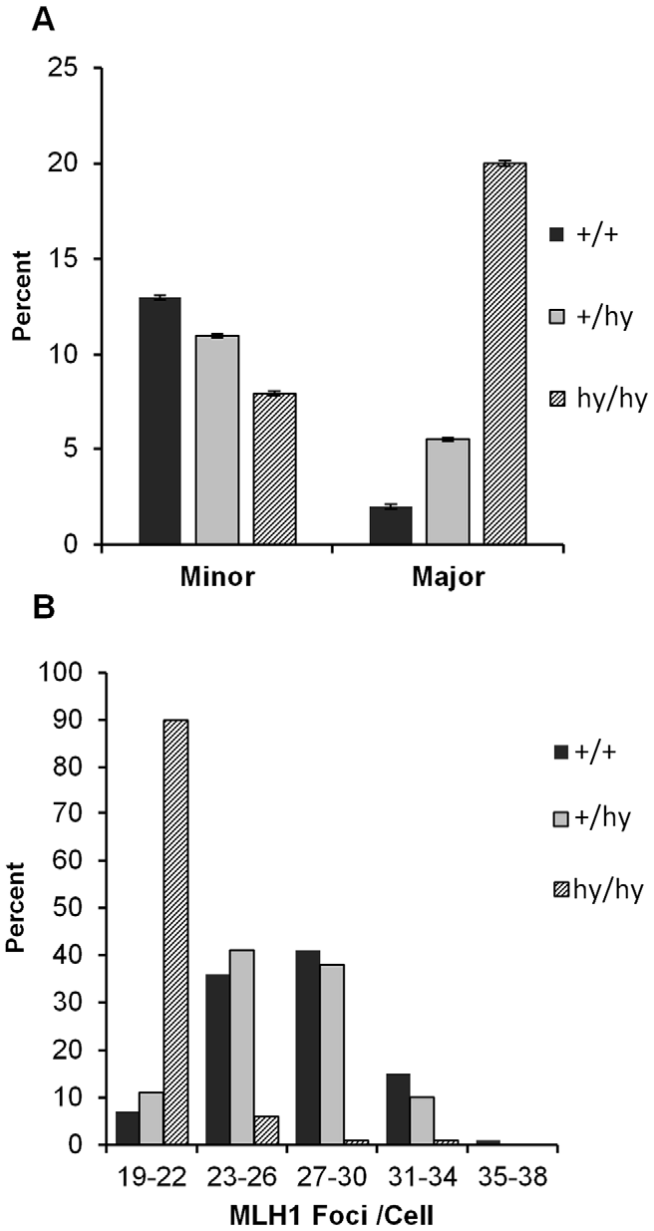
An *Smc1b* hypomorphic mutation affects synapsis and recombination. (A) Proportion of cells (± SE) with apparently normal synapsis, minor, or major defects. Although levels of synaptic defects were similar in wild-type and heterozygous females (+/hy), a marked increase in major defects was evident in homozygotes for the *Smc1b* hypomorphic allele (hy/hy); this produced a highly significant among-group difference in the frequency of synaptic defects (χ^2^_2 df_ = 35.7; *p*<0.0001). (B) Similarly, mean genome-wide MLH1 values ± SE were markedly decreased in homozygotes (20.7.9±0.21) by comparison with controls and heterozygotes (27.3±0.32 and 26.5±0.33, respectively) (F = 118.2; *p*<0.0001). These data represent 101 cells from 4 hypomorphic females (hashed bars), 100 cells from 5 hypomorphic heterozygotes (grey bars) and 100 cells from 4 wild-type siblings (black bars).

We recently used homologous recombination to generate a hypomorphic allele of *Smc1b* (see Materials and Methods). An analysis of synapsis and recombination in oocytes from heterozygous and homozygous carriers of this mutation revealed a gene dosage-specific increase in major synaptic defects, with approximately ten-fold higher levels in homozygous carriers of the hypomorphic allele (Figure 4A; χ^2^_2 df_ = 35.7; *p*<0.0001). Similarly, MLH1 levels varied significantly among the three genotypes (F = 118.2; *p*<0.0001) but the effect was entirely due to homozygotes (Figure 4B).

### Haploinsufficiency for SMC1β or REC8 increases the incidence of chromosomally abnormal eggs

**Figure 5:**
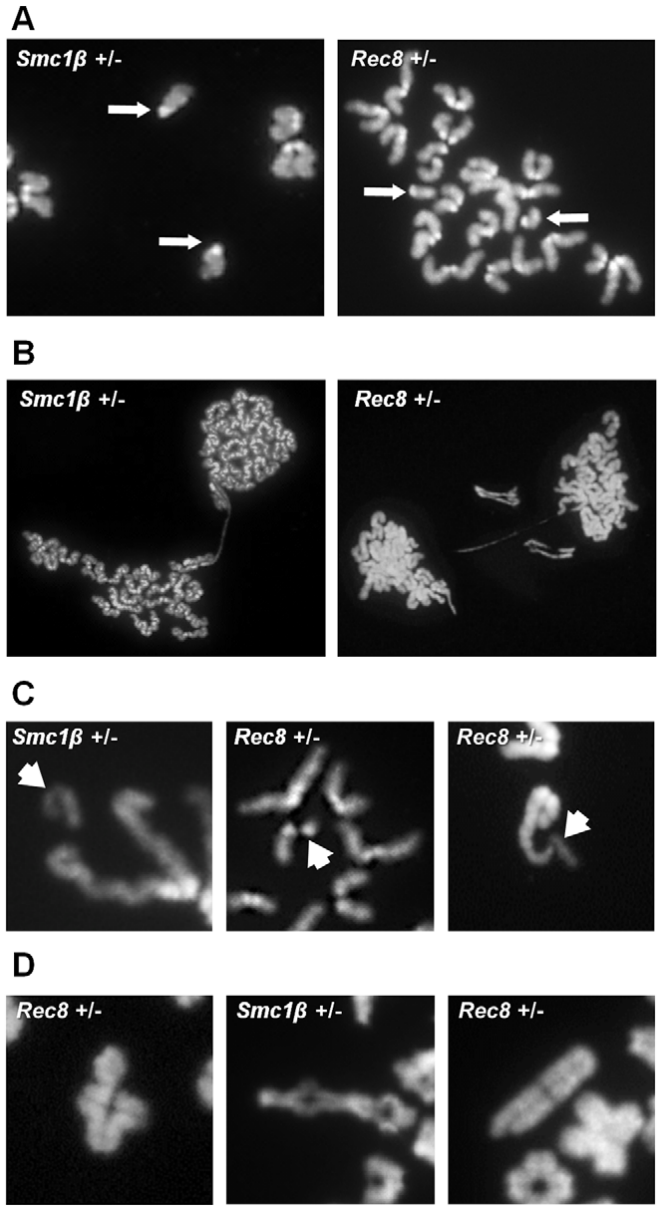
Chromosome abnormalities are increased in cohesin heterozygotes. Representative images of numerical and structural defects in cohesin heterozygotes. (A) Premature sister chromatid separation in MII eggs; arrows denote the unattached single chromatids. (B) Anaphase bridges between MII eggs and first polar bodies. (C) Chromatid defects in MII eggs; arrows denote abnormal chromatid. (Left) acentric fragment; (middle) deletion of almost an entire chromatid; (right) proximal chromatid break; note that acentric fragment remains attached to telomere of intact sister chromatid. (D) Defects in diakinesis stage oocytes: (left) bivalent with a chromatid break in one chromosome; (middle) nonhomologous end-to-end chromosome fusion that appears to involve both chromatids of two bivalents; (right) proximal break in a single chromatid.

Because alterations in the number and location of the sites of recombination are associated with meiotic nondisjunction [2] and the frequency of SCs lacking an MLH1 focus was increased in heterozygous carriers of both mutations, we analyzed metaphase II -arrested eggs to determine if defects induced during meiotic prophase affect the genetic quality of the eggs produced by heterozygous females. No difference was evident in the rate of germinal vesicle breakdown (meiotic resumption) or polar body extrusion (data not shown). However, the overall incidence of chromosomally abnormal eggs was 2–4 fold greater in heterozygotes than in wild-type controls (Table 3); for *Smc1b* heterozygotes, the increase reached statistical significance (χ^2^ = 4.0; *p*<0.05). Because of the relatively small series of eggs that we were able to analyze, it was not possible to determine whether statistically meaningful differences existed between heterozygotes and wild type controls for specific categories of abnormality. Nevertheless, it is notable that the increases in chromosomally abnormal eggs in heterozygotes resulted primarily from premature sister chromatid separation (PSCS) (Figure 5A). For *Smc1b* heterozygotes, 22.2% (10 of 45 eggs) exhibited PSCS by comparison with 3.7% (1 of 27 eggs) in wild-type siblings; similarly, in *Rec8* heterozygotes, 10% (9 of 90 eggs) exhibited PSCS by comparison with 6% (2 of 33 eggs) in wild-type siblings (Table 3).

**Table 3:** Chromosome abnormalities from cohesion heterozygotes and wild-type siblings.

Female				
	*Smc1b* +/+	*Smc1b* +/−	*Rec8* +/+	*Rec8* +/−
**Diakinesis/MI**				
Total Cells	37	72	57	48
Normal	37 (100.0%)	68 (94.4%)	57 (100.0%)	46 (95.8%)
Univalents	0	1 (1.4%)	0	0
St. Abnormal*	0	3 (4.2%)*	0	2 (4.2%)
**MII Arrested eggs**				
Total Cells	27	45	33	90
Normal	25 (92.6%)	33 (73.3%)	31 (93.9%)	78 (86.6%)
Hyperploid	1 (3.7%)	0	0	1 (1.1%)
PSCS**	1 (3.7%)	10 (22.2%)	2 (6.0%)	9 (10.0%)
St. Abnormal	0	2 (4.4%)	0	2 (2.2%)

Male				
	*Smc1b* +/+	*Smc1b* +/−	*Rec8* +/+	*Rec8* +/−
**Diakinesis/MI**				
Total Cells	42	71	42	34
Normal	42 (100.0%)	65 (91.5%)	42 (100.0%)	29 (85.3%)
Univalents	0	6 (8.5%)	0	5 (14.7%)
St. Abnormal	0	0	0	0
**MII**				
Total Cells	25	51	25	24
Normal	25 (100.0%)	51 (100.0%)	25 (100.0%)	23 (95.8%)
Hyperploid	0	0	0	0
PSCS	0	0	0	0
St. Abnormal	0	0	0	1 (4.2%)

* Structural abnormalities (MI abnormalities include 3 cells with chromatid breaks, 1 cell with a chromosome break, and 1 cell with an end-to-end fusion between two bivalents. All MII abnormalities were chromatid breaks).

** Premature sister chromatid separation.

Unexpectedly, our analysis also suggested an increased incidence of chromatid breaks and anaphase bridges in heterozygotes: 4.4% (2 of 45) of eggs from *Smc1b* and 2.2% (2 of 90) of eggs from *Rec8* heterozygotes (Figure 5, Table 3). By comparison, structural abnormalities were not detected in eggs from wild-type controls. Subsequently we analyzed oocytes at the diakinesis/MI stage to determine if structural aberrations were evident before the onset of anaphase I. Indeed, both *Smc1b* and *Rec8* heterozygotes exhibited low but elevated levels of structurally abnormal chromosomes by comparison with controls: 5.6% (4 of 72) of oocytes from *Smc1b* and 4.2% (2 of 48) of oocytes from *Rec8* heterozygotes exhibited aberrations (Table 3). All but one of these aberrations involved a break in a chromatid or a whole chromosome, and the remaining aberration was an end-to-end fusion between nonhomologous bivalents. Similar aberrations were not observed in 94 oocytes analyzed from control females. Although structural abnormalities were only observed in oocytes and eggs from heterozygotes, the difference did not reach significance for either mutation. However, because it is inherently difficult to obtain analyzable chromosome preparations from single oocytes and eggs, this presumably reflects the small number of cells analyzed.

To determine if numerical or structural chromosomal aberrations were also a feature of male meiosis, we analyzed MI and MII stage spermatocytes from heterozygotes. An increased frequency of univalents was evident at MI in both *Smc1b* and *Rec8* heterozygotes; for *Smc1b*, 8.5% (6 of 71) in heterozygotes and 0% (0 of 42) in wild-type males (χ^2^_1 df_ = 3.7, *p*<0.05), and for *Rec8* 14.7% (5 of 34) in heterozygotes and 0% (0 of 42) in controls (χ^2^_1 df_ = 6.6, *p*<0.02). However, a corresponding increase in hyperploid cells was not observed among MII spermatocytes (Table 3). In addition, out of a total of 180 MI and MII cells scored, only a single cell with a structural abnormality was observed. This suggests that, although chromosome abnormalities also occur in male heterozygotes, most are eliminated during the meiotic divisions.

### SMC1β protein levels also determine meiotic phenotypes during spermatogenesis

**Figure 6:**
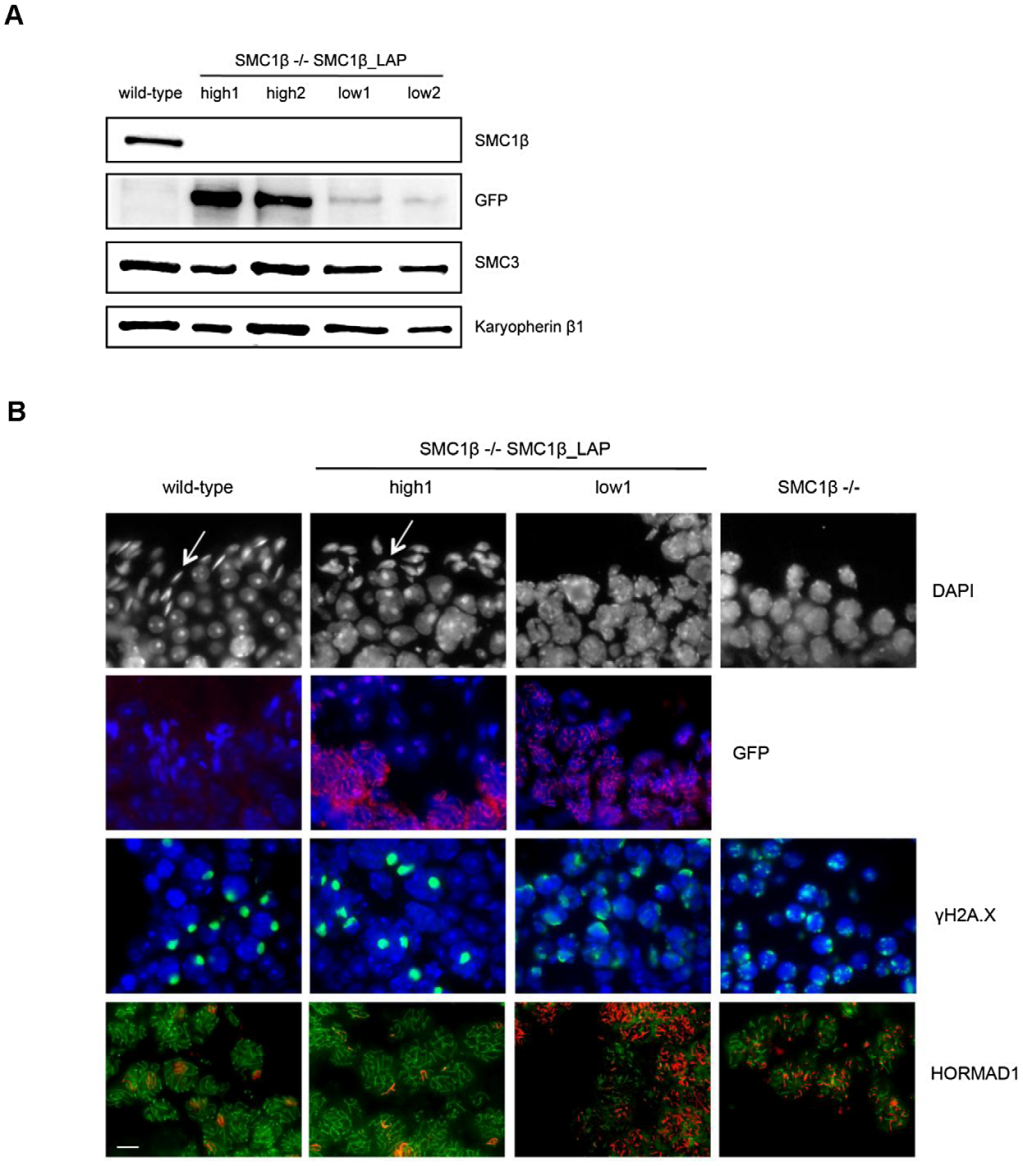
The effect of variable levels of SMC1β-LAP expression during meiosis.

To generate an additional model for varying levels of cohesin subunits during meiosis, we took advantage of mice carrying an *Smc1b*-Localization and Affinity Purification “LAP” BAC construct on an *Smc1b^−/−^* background [20]. The abundant and easily accessible material available from males provided the most efficient means of comparing protein levels in different individuals, and we screened multiple *Smc1b^−/−^* males carrying the construct and compared the meiotic phenotype of males with high and low protein levels (Figure 6A). Importantly, tagged protein localizes to chromosomes during meiotic prophase, suggesting it can fulfill its normal physiological role (Figure 6B). In *Smc1b* null males, cells become arrested in early/mid-pachytene, stage IV [18], and males with low SMC1β -LAP protein levels exhibited an indistinguishable meiotic phenotype. However, in males with higher protein levels (although still somewhat lower than wild-type), the *Smc1b* null phenotype was rescued, with cells completing meiosis as evidenced by the presence of elongated spermatids. To assess homolog synapsis, we examined the staining patterns of γ-H2A.X and HORMAD1. In wild-type males, γ-H2A.X typically forms a single distinct focus of staining around the predominately unsynapsed X and Y chromosomes, but also localizes to unsynapsed areas of autosomes, if present. In high SMC1β -LAP expressing cells, a strong single focus of γ-H2A.X sex body staining was evident, with proportions similar to wild-type cells (Figure 6B). In contrast, low expressing cells exhibited multiple foci per nucleus in an irregular pattern, similar to that observed in *Smc1b* null males [18]. HORMAD1, which localizes to unsynapsed regions [26], was indistinguishable from wild-type in males with high levels of SMC1β -LAP protein, suggesting normal synapsis. However, in males with low protein levels, HORMAD1 was consistently found on multiple chromosomes per nucleus, indicating the presence of asynaptic regions and mirroring the phenotype of *Smc1b* ^−/−^ cells. Although these studies suggest that rescue of the null phenotype depends on the levels of SMC1β -LAP protein produced, subtle meiotic differences may exist between these males and wild-type mice, as well as between the SMC1β -LAP low expressers and *Smc1b* ^−/−^ cells.

## Discussion

Recent data from a variety of sources support the hypothesis that, in the mammalian oocyte, meiotic cohesion weakens over time, and loss of cohesion contributes to meiotic errors that cause aneuploidy [10]. This prompted us to ask whether heterozygosity for mutations in meiosis-specific cohesin genes might also elicit meiotic effects in mammals. The data presented here using four different mouse models provide evidence that altered gene dosage for either of the meiosis-specific cohesins, *Rec8* or *Smc1b* adversely impacts the events of meiotic prophase during both oogenesis and spermatogenesis. These findings are consistent with a recent report on a somatic chromosome instability phenotype in mice heterozygous for the centromeric cohesin protector Shugoshin-1 (Sgol1) [27]. Enhanced tumorigenesis following carcinogen exposure and increased chromosome mis-segregation in mouse embryonic fibroblasts were observed. Given the data presented in our report it seems likely that the role of Sgol1 in cohesin protection and not some other, unknown function of shugoshin caused these phenotypes.

Our analyses of heterozygous carriers of mutations in either *Rec8* or *Smc1b* and of homozygous carriers of a hypomorphic allele of *Smc1b* demonstrated defects in both synapsis and recombination. These findings suggest that appropriate dosage of each cohesin subunit is essential for these events. Consistent with this interpretation, analyses of *Smc1b* null males carrying either a “high” or “low” expressing SMC1β transgene revealed strikingly different meiotic phenotypes. Specifically, carriers of the low expressing transgene exhibited a meiotic phenotype virtually identical to the SMC1β “knockout” [18], with no cells progressing beyond mid-pachytene. In contrast, in males carrying the high expressing transgene, normal synapsis was restored, the sex body was indistinguishable from wild-type, and elongating spermatids were present, indicating cells were able to progress beyond pachytene. Taken together, our data provide compelling evidence that the correct dosage of meiotic cohesins is essential for normal meiotic prophase.

Importantly, the subtle prophase changes induced by altered cohesion gene dosage have a significant impact on gamete quality. As reported for other meiotic mutations [28]–[30] and for environmental effects that alter synapsis and recombination [31], the synaptic defects and alterations in the number and placement of recombination events observed at prophase increased the likelihood of meiotic chromosome segregation errors. However, unlike other meiotic mutations, our results suggest that reductions in cohesin components may increase the likelihood of structural, as well as numerical, chromosome abnormalities.

The harlequin appearance of SCs, particularly near the chromosome ends, and the occurrence of anaphase bridges in oocytes are consistent with the previous report of an increase in telomere defects in SMC1β knockout animals [32]. However, our results extend the hypothesis that SMC1β plays an essential role in the protection of meiotic telomeres, suggesting a more general effect of cohesin dosage. That is, in the majority of oocytes in which structural abnormalities were identified, the breaks occurred interstitially or in pericentromeric regions, not at telomeres (e.g., Figure 5C right and 5D left). This suggests that cohesins play a role in protecting not only telomeres, but all of the DNA within the context of the synaptonemal complex. The most obvious explanation is that the observed breaks represent sites of unrepaired DNA damage, due either to a delay in repair imposed by defects in synapsis or to impediments in DNA repair as recently reported for Rad21 [33]. However, in addition to single chromatid breaks that would be predicted to result from failure to complete repair at the site of a double strand break, we also observed breaks involving both chromatids in a small number of cells. Thus, it is possible that the DNA held within the altered SC scaffold may somehow be rendered vulnerable to damage during late prophase, after the repair of programmed double strand breaks is complete. This raises obvious questions for future studies; e.g., does altered gene dosage for meiosis-specific cohesins sensitize meiocytes to DNA damaging agents and do allelic variants play a role in the generation of human structural chromosome abnormalities?

Further, our findings in mice have important implications for humans. The incidence of aneuploidy in humans is astonishingly high and the magnitude of the effect of advancing maternal age makes it difficult to discern effects of other, more subtle, causal agents. Nevertheless, it has been postulated that some individuals may be prone to nondisjunction because they carry mutations in meiotic genes [34]. Our studies in mice suggest that this is the case for carriers of mutations or variants that affect meiosis-specific cohesin gene dosage. Importantly, our data suggest that the increases might not be limited to numerical abnormalities but could also include structural rearrangements, and that this would be evident even at young maternal ages.

Our analyses suggest cohesin dosage effects in both male and female mice. Does this mean that, in humans, the likelihood of producing chromosomally abnormal gametes may be increased for both men and women who carry mutant or variant alleles of cohesin genes? Weakened cohesion would be expected to lead to both premature loss of the cohesion that holds chiasmata in place – yielding univalent chromosomes at MI - and to premature separation of sister centromeres – yielding single chromatids at either MI or MII. Our analysis of *Rec8* and *Smc1b* heterozygotes confirm the occurrence of both univalents and single chromatids (Table 3, Figure 5A). Further, previous studies by us and others (e.g., [7], [35], [36]) demonstrate a straightforward correlation between the presence of univalents and/or single chromatids and the production of aneuploid eggs. Thus, abnormalities in cohesion are attractive candidates for female-derived aneuploidies. However, although univalents and single chromatids are frequently able to evade meiotic spindle assembly checkpoint control mechanisms in mammalian females [37], in males the checkpoint is extremely stringent [38]. Thus, in the male, aberrant chromosomes caused by cohesion deficiencies would be expected to cause metaphase arrest and cell death, possibly reducing sperm numbers. However, they would not be expected to appreciably increase the frequency of aneuploid sperm. This expectation is confirmed by the results of our studies of male heterozygotes: the frequency of univalents at MI was significantly increased in both *Rec8* and *Smc1b* heterozygotes, but a corresponding increase in aneuploidy was not evident at MII (Table 3), suggesting effective elimination of cells with univalent at MI.

Our analyses also suggested an increase in chromosome breaks prior to, or at the onset of, anaphase I in females. We observed a range of different structural aberrations in oocytes and eggs from female heterozygotes, including anaphase bridges, breaks near centromeric regions, nonhomologous fusions, and different types of interstitial breaks (Figure 5). These findings suggest that carriers might be at increased risk of transmitting different types of aberrations, including Robertsonian fusions and other reciprocal translocations, deletions, and duplications. Whether structural aberrations would also be transmitted by male carriers almost certainly depends upon their origin. That is, based on our understanding of sex-specific differences in cell cycle control, the expectation is that spermatocytes with unrepaired double strand breaks would be effectively eliminated during prophase [38]. In contrast, breaks induced just prior to or during the meiotic divisions would not be expected to trigger cell death, since the presence of sticky chromosome ends or fragments at metaphase should not interfere with spindle attachment. Similarly, since anaphase bridges are manifested after cell division is initiated, they do not trigger cell cycle arrest [39]. Thus, the fact that structural aberrations were comparatively rare in spermatocytes at MI and MII by comparison with comparable stages in the female supports the hypothesis that most breaks result from the presence of a small number of unrepaired double strand breaks.

In summary, our results suggest that proper orchestration of meiotic prophase in mammals requires accurate dosage of meiosis-specific cohesin genes and that women who are asymptomatic carriers of mutations or polymorphic variants in meiosis-specific cohesins may be at increased risk of producing chromosomally abnormal gametes. Specifically, we hypothesize that women who carry a functionally impaired allele have an increased risk of aneuploid pregnancies even at young maternal ages and may even be at increased risk of transmitting structural abnormalities. For males, however, our findings suggest that, because of stringent meiotic cell cycle control mechanisms, most numerical errors and structural rearrangements would be prevented from contributing to the population of viable gametes.

## Materials and Methods

### Ethics statement

All animal experiments were approved by the WSU Institutional Animal Care and Committee and conducted in accordance with the Guide for Care and Use of Laboratory Animals.

### Animals

The *Smc1b* and *Rec8* mutants used in this study have been described previously [18], [19]. The *Smc1b* mutation removes exon 10, which codes for approximately 40% of the hinge domain necessary for the formation of the SMC heterodimer that is essential for formation of the cohesin complex function. In *Smc1b* ^−/−^ mice, *Smc1b* gene expression is dramatically reduced, and SMC1β protein is not detectable by either immunoblotting or immunofluorescence [18]. The *Rec8* mutation has removed 19 of the 20 coding exons of the gene and functional transcripts are not detectable in *Rec8* ^−/−^ mice [19]. To identify wildtype and heterozygous females for these studies the genotypes were determined by PCR amplification of DNA as described previously [18], [19].

The hypomorphic allele of *Smc1b* was created during the construction of the loxP-SMC1β described previously [20]. The mutant allele retains the *neo* gene but in reverse orientation. Genotyping was performed as described previously [20] using primers 1, 2 and a new primer (CAC GCG TCA CCT TAA TAT GC) designed for the reversed neo cassette with a product of ∼520 bp for the hypomorphic allele. The mutation is on a mixed genetic background, but experiments were done using animals from second or third generation backcrosses to C57BL/6. Homozygous hypomorphic males are sterile (2 males, 6 months) and females are subfertile (3 females, 8 litters at ∼2pups/litter, 6 months).

The *Smc1b*-LAP BAC construct was generated by recombineering [40] using a BAC carrying the genomic locus (ENSG00000077935) and the R6Kamp-hNGFP plasmid to generate an N-terminally tagged *Smc1b* gene within the natural gene expression control elements. The *Smc1b*-LAP BAC was injected into blastocysts, and five founders were obtained and mated with animals carrying the *Smc1b* mutation.

### Meiotic analyses

For analyses of synapsis and recombination, surface spread preparations were made from 18 dpc fetal ovaries and testes from 8-week old males [41]. Slides were immunostained, examined on a Zeiss epifluorescence microscope, imaged with a CCD camera, and analyzed using Axiovision software. Pachytene stage cells were identified on the basis of synaptonemal complex (SC) morphology [42], and all cells were analyzed by two independent observers who were blinded with regard to the genotype of the animals. To calculate the frequency of synaptic defects, 50 pachytene cells per animal were scored for the presence of minor or major defects. If one or more SC exhibited a gap (identified as a small discontinuity in localization of the SC protein SYCP3), a fork (a separation of the telomeric region of the SC, comprising no more than one third of the overall length of the SC), or a bubble (an internal separation of the SC comprising no more than one third of the length of the SC) the cell was categorized as having minor defects. The cell was categorized as having a major defect if one-third or more of the length of an SC was asynapsed. To analyze recombination, slides were immunostained with antibodies to MLH1 (Calbiochem, Millipore MA, USA) and SYCP3 (Santa Cruz Biotechnology, Inc., CA, USA). The total number of MLH1 foci per cell was determined for at least 25 cells per individual and for at least five animals of each genotype. MLH1 foci were scored only if the signals were punctate in appearance, localized on the SC, and separated from adjacent foci by at least one signal domain. For studies of SC morphology, slides were immunostained with antibodies to both SYCP1 (Santa Cruz Biotechnology, Inc., CA, USA) and SYCP3 and the appearance of the SC was examined for 50 pachytene cells per genotype.

### Cytogenetic analysis of diakinesis/MI and MII cells

Chromosome preparations were made from oocytes collected from 28-day old females and matured *in vitro* as described previously [43]. Briefly, oocytes were collected and cultured in Waymouth medium (Gibco, Invitrogen Carlsbad, Ca, USA) supplemented with 10% fetal bovine serum and 0.23 mM sodium pyruvate. After two hours in culture, oocytes that remained at the germinal vesicle stage were removed, and the remaining oocytes were cultured for 4 hrs or overnight (∼12–14 hrs) to obtain MI and MII eggs, respectively. After culturing, eggs were treated in a hypotonic solution (0.9% sodium citrate), fixed onto slides with 3∶1 methanol∶acetic acid, and stained with DAPI. Metaphase images were captured with a CCD camera on a Zeiss epifluorescence microscope and analyzed by two independent observers who were blinded with respect to genotype. For the analysis of MI and MII stage spermatocytes, air dried preparations were made as described by Evans [44] and the same capturing and blind scoring methodology used.

### Cryosectioning and Western analysis of testes from *Smc1b*-LAP males

Whole testes were immersed in O.C.T Compound (Tissue-Tek 4583) in specimen molds (Tissue-Tek 4566 Cyromold 15 mm×15 mm×5 mm) and frozen at −80°C. 7 µm sections were cut using a Leica CM1900 and placed on microscope slides (StarFrost K078; 76×26 mm). Sections were fixed using 4% formaldehyde (Sigma F8775) in 1× PBS for 15 mins at 22°C and permeablised using 0.15% Triton-X100 (Servas 37240) for 10 mins at 22°C and washed twice in 1× PBS. Slides were blocked in 2% BSA (Sigma A2153) in 1× PBS for 30 mins and primary antibodies were incubated at 4°C for 16 hours. Primary antibodies: anti-SYCP3 mouse monoclonal, hybridoma cell line supernatant (1∶1, kind gift from Christa Heyting), anti-γ-H2A.X phospho-ser139 (1∶600, mouse monoclonal IgG_1_, Millipore 05-636), anti-eGFP (1∶500, goat, MPI Dresden), anti-Hormad1 (1∶700, guinea-pig, kind gift from A. Tóth, Dresden). Slides were washed 3× in 1× PBS and secondary antibodies were incubated for 1–2 hours at 22°C. Slides were washed 3× in 1× PBS and mounted using VectaShield mounting media (Vecta Laboratories, H-1000) plus 1 µg/µl DAPI and 24×50 mm coverslips (Engelbrecht, K12450, depth 0.13–0.17 mm). Testes sections were imaged using a Leica Axiophot microscope at 100× or 40× magnification with oil of refractive index 1.518 (Zeiss, Immersol 518 F).

For protein extraction and Western blotting, the tunica albuginea was removed from the testes and a single cell suspension created using Dounce homogenisation (loose pestle) in Buffer B (5 mM KCl, 2 mM DTT, 40 mM Tris.HCl (pH 7.5) 2 mM EDTA, 0.5 mM spermidine and protease inhibitors) followed by Dounce homogenisation (tight pestle). The cell suspension was centrifuged at 8000 rpm for 3 mins, the nuclear pellet resuspended in Buffer C (5 mM KCl, 1 mM DTT, 15 mM Tris.HCl (pH 7.5), 0.5 mM EDTA, 0.5 mM spermidine and protease inhibitors), 250 mM ammonium sulphate (pH 7.4) was added and incubated on ice for 30 mins. Samples were centrifuged at 45,000 rpm for 30 mins at 4°C. Supernatant was collected and protein content measured by Bradford before being stored at −20°C in Laemmni buffer for Western analysis. 5 µg of protein was run on a 6% SDS-PAGE gel, transferred to a nitrocellulose membrane, and blocked in 5% milk in PBST (PBS plus 0.1% Tween-20) for 1 hour at 22°C. Primary antibodies were added at 1 µg/µl in 5% milk in PBST for 16 hours at 4°C. Primary antibodies: anti-karyopherin β1 (H-300) (sc-11367 rabbit polyclonal IgG Santa Cruz), anti-eGFP (goat, MPI Dresden) and anti-SMC3 (A300-060A, rabbit, Bethyl). Blots washed 3× in PBST and HRP-conjugated secondary antibodies were added for 1 hour at 22°C in 5% milk in PBST. Blots were washed 3× in PBST and developed using chemiluminescent HRP substrate (Millipore, WBKLS) and imaged on a Kodak ImageStation 2000MM.
